# The temporal dynamics of the association between daily physical activity and life satisfaction

**DOI:** 10.1093/abm/kaaf079

**Published:** 2025-11-11

**Authors:** Steriani Elavsky, Marek Brabec, Marek Maly, Lenka Knapova, Barbora Kastovska, Michal Sebera, Marcela Ely, Vera K Jandackova, Jan Keller, Misha Pavel

**Affiliations:** Department of Human Movement Studies, Faculty of Education, University of Ostrava, Ostrava, 70200, Czech Republic; Institute of Computer Science, The Czech Academy of Sciences, Prague, 18200, Czech Republic; Institute of Computer Science, The Czech Academy of Sciences, Prague, 18200, Czech Republic; Department of Human Movement Studies, Faculty of Education, University of Ostrava, Ostrava, 70200, Czech Republic; Department of Human Movement Studies, Faculty of Education, University of Ostrava, Ostrava, 70200, Czech Republic; Department of Human Movement Studies, Faculty of Education, University of Ostrava, Ostrava, 70200, Czech Republic; Department of Human Movement Studies, Faculty of Education, University of Ostrava, Ostrava, 70200, Czech Republic; Department of Human Movement Studies, Faculty of Education, University of Ostrava, Ostrava, 70200, Czech Republic; Heidelberg University, Heidelberg, 69117, Germany; Freie Universität Berlin, Berlin, 14195, Germany; Department of Human Movement Studies, Faculty of Education, University of Ostrava, Ostrava, 70200, Czech Republic; Khoury College of Computer Science, Northeastern University, Boston, MA, 02115, United States

**Keywords:** life satisfaction, physical activity, exercise identity, dynamic modeling, within-person variability, Bayesian vector autoregressive model, air pollution

## Abstract

**Purpose:**

Life satisfaction (LS) is increasingly recognized as a crucial indicator and predictor of health and well-being across the lifespan. The impact of LS may be enhanced through physical activity (PA), although studies exploring the dynamic and bidirectional nature of the relationship are scarce. One principal goal of this project is to examine the dynamic, personalized interactions between LS and PA and exercise identity (the degree to which exercise is a fundamental aspect of one’s self-concept) in geographic areas with different air pollution loads.

**Method:**

We used data from a 12-month prospective cohort study (*N* = 1314, mean age = 38.09 [12.55]; range 18-65) with four 2-week intensive measurement bursts to evaluate the bidirectional relationship between LS (assessed at the end of the day) and PA (assessed by Fitbit Charge 3 or 4 throughout the day). The sample included both active (runners; *n* = 747, 57%) and inactive (*n* = 567, 43%) individuals living in Moravia-Silesia and South Bohemia, geographic areas with different levels of air pollution. A dynamic Bayesian model based on an extension of the vector autoregressive model was used to estimate both lagged and contemporaneous associations between LS and PA.

**Results:**

There were meaningful autoregressive effects of first order for both LS (β = 0.394) and PA (β = 0.316), and a within-person contemporaneous association between LS and PA (β = 0.087) that was also associated with temporal factors and trends (weekly and monthly seasonal variation, day in study), gender, age, and exercise identity.

**Conclusion:**

This study highlights the importance of periodicity on 2 temporal scales for both PA and LS, with age and gender also playing crucial roles. The findings underscore the importance of tailored, context-aware interventions to sustain engagement and enhance well-being through PA.

## Introduction

Life satisfaction (LS) is increasingly recognized as a crucial indicator of health and well-being, with substantial evidence supporting its role in enhancing various health outcomes. As a global indicator of subjective well-being, LS is now included alongside socioeconomic indicators such as GDP in national well-being indices.^1^^,^^2^ Research also indicates that LS represents a valuable target for health-related policies and interventions because it is associated with better physical health,^3^ including lower risks of pain,^4^ physical functioning limitations, and mortality,^5^ as well as fewer chronic conditions and better self-rated health.^3^^,^^6^^,^^7^ While most evidence supports poor physical health predicting lower LS, the reverse direction remains less consistently supported in longitudinal investigations.^8^^,^^9^

One way to enhance both health and LS is through physical activity (PA), which has been shown to have multiple physical and mental health benefits.^10^ Indeed, there is growing evidence supporting the links between LS and PA.^11^^,^^12^ For example, a recent review focused specifically on the links between PA and happiness (LS being the most commonly used measure of happiness) concluded that as little as 10 minutes of PA per week, or 1 day of doing exercise per week, significantly impacts people’s happiness level.^12^ Existing research, however, predominantly relies on static (cross-sectional) or infrequent assessments. Such study designs are inherently limited not only in establishing causality but also in their ability to capture the dynamic and potentially bi-directional nature of the relationship between PA and LS (ie, the situation in which past PA can influence current LS and also past LS can influence current PA). Prospective studies, which are far scarcer, can shed more light into temporal order and causality, but their infrequent assessments do not capture day-to-day variability or temporal patterns. The ascend of smartphone technology has enabled more frequent data capture in the context of intensive longitudinal designs (ILD),^13^^,^^14^ such as daily diary studies or ecological momentary assessment (EMA), allowing researchers to explore both within- and between-person variability in real time for both well-being^15^ and health behaviors, including PA.^16^

### Life satisfaction and physical activity: a dynamic relationship

Existing ILD studies concretely show that day-to-day variations in PA correlate with fluctuations in LS, suggesting that the PA-LS link is not only a function of individual differences (ie, people with high PA reporting higher LS than those with low PA levels) or long-term effects (a “top-down” mechanism), but also that short-term fluctuations in PA influence daily assessments of LS (a dynamic “bottom-up” mechanism).^17–20^ A recent systematic review of 53 smartphone-based EMA studies has demonstrated that well-being fluctuates daily and weekly and that PA and other environmental variables contribute to this fluctuation.^15^ In support of this point, Maher et al^21^ have shown in 2 studies of emerging adults across 8 and 14 days, respectively, that higher LS was reported on days when participants engaged in more PA than was typical for them, while controlling for other competing influences (eg, gender, body mass index [BMI], and other personality and psychological variables). Later, in a study involving both young and older adults, Maher et al^19^ have shown that this within-person association between PA and LS held true, irrespective of age. In a 4-day study of adolescents, Bourcke et al^22^ have similarly shown that on more active days, adolescents reported higher LS, with energetic arousal mediating the relationship. These findings were replicated with objective measurement of PA across 14 days, also demonstrating that sedentary behavior has an additive, negative effect on daily LS in both emerging adults^23^ and older adults.^24^ It should be noted that most existing studies have explored the PA and LS relationship at the contemporaneous level, with limited evidence on lagged associations between the 2 variables (as in van Woudenberg et al^25^). A recent study based on 7 days of GPS data and activity diary data has additionally demonstrated a positive correlation between GPS trajectories-derived city space data (namely, air pollution, city vibrancy, physical urban form) and activity satisfaction, with both day-to-day and hour-to-hour periodicity effects that predicted activity satisfaction levels,^26^ highlighting the importance of contextual influences on subjective well-being.

### Gaps in the literature and the need for contextualized analysis

In their systematic review, de Vries et al^15^ point out important knowledge gaps in the available literature. First, the majority of existing studies are short in duration (average study duration was 12.8 days), with only half of the studies including objective data on behavior or context. Although existing studies clearly demonstrate substantial daily and weekly variability in both PA and LS and the influence of environmental context,^15^ few studies with longer sampling periods and objective monitoring of PA exist. Among the main disadvantages, this precludes the possibility to account for seasonality in activity and possibly also LS patterns, which might be important at least in temperate climate zones. Failure to account for the annual (but also weekly) periodicity can lead to results that are limited to interpretation within a specific temporal, environmental, or psychological context (eg, strong motivation effects at the onset of a short-term study). For example, the PA-LS association may be genuinely stronger during summer months due to increased daylight and favorable weather and weaker during winter due to reduced opportunities for PA and seasonal affective symptoms. Thus, to gain a comprehensive understanding of the PA-LS association, research must employ designs that can account for and test these contextual moderators over time.^27–29^

Environmental factors also remain underexplored. Air pollution, for example, discourages outdoor activity and is independently linked to lower LS and poorer health outcomes.^30^^,^^31^ Yet, how environmental context, such as residing in more or less polluted areas, might moderate the PA-LS relationship remains underexplored. Additionally, many studies have relied on relatively homogeneous samples, underrepresenting variation by age, gender, education, or socioeconomic status (SES). These variables are crucial to understanding PA and LS, as both show systematic variation across demographic groups.^7^^,^^32^^,^^33^ For instance, older adults may report higher LS and more routine PA due to fewer competing obligations,^34^ while lower SES or education may limit access to health-promoting environments and opportunities for PA, and this can ultimately lead to lower LS.^7^ Another key limitation of existing studies is small sample sizes and homogeneity of the samples in terms of age and PA status, with the majority of studies involving small samples of either young or older adults and focusing on individuals with low levels of PA to begin with.^11^^,^^15^ This is problematic given the evidence that LS tends to change across the lifespan, being lower during emerging adulthood, higher during midlife, and lower during older adulthood.^19^^,^^32^ Similarly, focusing mostly on low-active individuals leaves a gap in understanding of how PA and LS may transpire in moderately and highly active individuals or those who place a high value on exercise.

Indeed, the importance of relative comparisons in LS (ie, how individuals evaluate their own LS by comparing themselves to others) has been previously highlighted, showing that subjective well-being is influenced not only by absolute levels of factors such as income and health but also by how these compare to personal reference points and expectations.^35^ Self-determination theory (SDT) posits that the quality of a person’s motivation significantly impacts behavior and well-being. According to SDT, individuals are more likely to engage in and sustain PA when they experience autonomous motivation, which includes intrinsic motivation (engaging in an activity for its inherent satisfaction) and identified regulation (recognizing the personal importance of the activity).^36^ Exercise identity, or the extent to which individuals see themselves as exercisers, can enhance autonomous motivation in the long term. When exercise is a core part of one’s identity, individuals are more likely to engage in PA consistently, driven by internal values and enjoyment rather than external pressures.^37–39^ This alignment with intrinsic goals can lead to higher LS, as the activity fulfills basic psychological needs for autonomy, competence, and relatedness.

Exercise identity is a specific aspect of self-concept that reflects how central PA is to an individual’s self-definition. A strong exercise identity can lead to greater commitment to PA, as individuals strive to maintain consistency between their behavior and self-concept.^38^^,^^40^ This consistency can enhance LS by providing a sense of purpose and coherence in one’s life. Moreover, individuals with a strong exercise identity may experience greater psychological benefits from PA, such as improved mood and self-esteem, further contributing to overall LS,^41^ or conversely, be more negatively impacted by declines in PA with respect to well-being.^42^ One could thus theorize that exercise identity could moderate the PA-LS relationship. For individuals with a strong exercise identity, PA may have a more pronounced impact on LS, as it aligns with their core values and self-concept. Conversely, for those with a weaker exercise identity, the relationship between PA and LS may be less robust, as the activity may not hold the same personal significance. Understanding this moderating role can help tailor interventions to enhance LS through PA by fostering a stronger exercise identity.

### The present study

This study addresses key limitations in the existing literature by investigating the temporal dynamics and factors implicated in the PA-LS relationship using a 12-month ILD design. Participants were drawn from 2 Czech regions differing in air pollution exposure (Moravia-Silesia and South Bohemia), and PA was assessed objectively via Fitbit devices. LS was measured through end-of-day EMA surveys across four 2-week bursts. We apply a dynamic Bayesian vector autoregressive (VAR) model to test both contemporaneous and lagged associations between PA and LS, while accounting for temporal factors (weekday, season, study day) and individual-level moderators, including age, gender, education, SES, air pollution exposure (via residency), and exercise identity. By integrating environmental, demographic, and psychological variables into the modeling of within-person processes, this study offers a comprehensive and context-sensitive perspective on how PA and LS interact across time and individual differences. Importantly, while demographic factors (eg, age, gender, SES, education) and environmental/contextual factors (eg, residency status as a proxy for air pollution, seasonality, weekday) were specified as covariates influencing the mean levels of LS and PA, exercise identity was tested as a moderator of the contemporaneous PA-LS association.

## Methods

### Study design

The 4HAIE study is a 12-month prospective longitudinal investigation designed to explore the links between air pollution, biomechanical, physiological, psychosocial, and sociodemographic variables, and their interaction on the incidence of running-related injuries, physical (in)activity, health, and quality of life among adults aged 18 to 65 years.^43^ The study’s interdisciplinary nature allowed for the integration of data from dynamic real-time monitoring of PA and psychosocial factors in a sample originating from 2 different contexts—areas with high (Moravia-Silesia) and low (South Bohemia) ambient air pollution status. It is well documented that these 2 areas differ both in annual mean concentrations (eg, based on the average of annual mean concentrations from 2017 to 2021) and in lifetime exposures to air pollutants such as PM_10_, PM_2.5_, NO_2_, benzene, and B(a)P.^44^^,^^45^ Although the study was conducted in 2 separate Czech regions, they are geographically close and climatologically similar. Both regions experience continental climates with minimal differences in temperature, daylight, and precipitation throughout the year. Furthermore, participants from both regions completed data bursts during the same calendar periods, controlling for seasonal exposure.

### Participants

The study was conducted with 1314 participants, recruited from the Moravian-Silesian Region (MSR, high air pollution; *n* = 750 or 57.1%) and the South Bohemian Region (SBR, low air pollution; *n* = 564 or 42.9%) of the Czech Republic. Given that one of the primary aims of the study was to establish a prospective cohort study for the assessment of running-related injuries, the recruitment strategy aimed to establish a sample stratified by age and PA status, with the goals of recruiting adults from the age of 18 to 65 and having 60% of the sample represented by active runners. The final distribution of the activity status of the sample at baseline was 747 runners (56.8%) and 567 inactive controls (43.2%).

Recruitment of participants was commissioned to a professional social science research and marketing company through a public tender process. The company used a variety of recruitment methods ranging from online recruitment (eg, social media posts, online fora, job websites), recruitment in communities and at community events (eg, sports clubs, mall stands), media ads (eg, local newspapers, local transit, radio), recruitment through agency interviewer network, and by chain referral. Participants were required to be non-smokers, have internet access on a smartphone, and have no physician-diagnosed restrictions to PA.

The eligibility criteria were assessed at baseline via an online screening survey, followed by telephone screening. PA status was assessed by self-report as part of the online screening survey. Active runners were participants who indicated meeting current recommendations for PA (ie, participating in 150 minutes or more in moderate to vigorous PA per week) and were participating in regular running (at least 10 km per week) in the past 6 weeks or longer. Inactive controls were participants who, upon screening, self-reported performing less than 150 minutes of moderate to vigorous PA per week but were capable of running. Given the EMA assessment, participants were required to have a smartphone (Android or iOS) with internet access. Participants without smartphone were offered a research smartphone for the duration of the study. Exclusion criteria included smoking (to rule out preexisting negative health effects of smoking), pregnancy (due to safety and ethical considerations), acute illness (due to safety considerations for baseline fitness/biomechanics testing), contraindications to magnetic resonance imaging, or conditions limiting PA.^43^^,^^46^^,^^47^

### Data collection procedures

The study employed an EMA protocol, which involved intensive survey data collection using a mobile app during four 2-week measurement bursts over the 12-month period. Participants entered the study continuously, with baseline assessments occurring between April 2019 and August 2021, and the EMA data collection for the whole study taking place between April 2019 and August 2022 as part of the 12-month monitoring period. For each participant, burst 1 occurred during weeks 1 and 2 of the study, burst 2 during month 4, burst 3 during month 8, and burst 4 during month 12. EMA offers the opportunity to assess instantaneous subjective states with short questions. Participants were prompted to respond according to a semi-random schedule to minimize adaptation to a predictable schedule and resulting artifacts, with semi-random sampling within fixed intervals: 8:00-11:59, 12:00-15:59, 16:00-19:59, and 20:00-22:00. The study uses ratings of LS completed as part of the evening survey. The study procedures were approved by the Ethics Committee at the University of Ostrava (No. OU-22953/90-2020), and participants provided written informed consent prior to data collection, adhering to the standards set by the Declaration of Helsinki.

### Measures

*Sociodemographics.* Information about age, gender, education level, and SES was ascertained from baseline online survey (between 2019 and 2021). Education was rated in 5 categories, but for the purposes of analysis, the variable was dichotomized into university education vs non-university education (ie, category 5 vs others in Table 1). In terms of SES, participants answered the following question “How would you rate your family’s economic situation” using the following answer options: 1—below average, 2—average, 3—above average. During recruitment and subsequent screening, participants’ residency status was determined (MSR vs SBR).

**Table 1.** Sociodemographic descriptive statistics of the sample.

Variable	% missing	Category	Proportion of *N* = 1314 *M* (SD) or % (*n*)
**Age**		Range: 18-65	38.09 (12.55)
**Gender**		Women: 46.3% (608)	46.3% (608)
**Location**		Moravian-Silesian Region	57.1% (750)
**Activity status**		Runners	56.8 (747)
**Education**	1.3% (17)	Primary (including unfinished)	4.8% (63)
		Apprentice, secondary vocational without certification	7.8% (103)
		Secondary school diploma (general and vocational)	40.2% (528)
		Higher vocational (post-secondary, DiS)	2.9% (38)
		University and higher (Bc, MSr, Ing, PhD)	43.0% (565)
**SES**	1.6%	Below average	2.5% (33)
	(21)	Average	78.5% (1032)
		Above average	17.4% (228)

Abbreviations: *M*, mean; SD, standard deviation; SES, socioeconomic status.

*Life satisfaction.* LS was assessed as part of the evening survey administered between 20:00 and 22:00 using 2 items from the Satisfaction with Life Scale by Diener et al^48^: “In most areas, my life today was close to my ideal” and “Today I am satisfied with my life.” Each item was rated on a slider scale ranging from 0 “disagree” to 100 “agree.” We averaged the 2 items to get LS variable in our analysis.

*Physical activity.* Participants were monitored across 12 months by a Fitbit Charge 3 (or Charge 4 for participants in the later parts of the study due to unavailability of Charge 3 versions to replace faulty devices) and were required to wear the monitor all day, including sleep. An exception to wearing the Fitbit was when there was a potential risk of injury (eg, during contact sports or in sauna). The Fitbit Charge 3 and Charge 4 used in this study share nearly identical hardware and step-counting algorithms. The primary distinction is the inclusion of built-in GPS in Charge 4, which was not used in our analysis. Both devices provide minute-level step counts, the basis for our PA measure. Validation studies^49^^,^^50^ support their comparability in step tracking. Altogether 63 participants (4.8%) wore Fitbit Charge 4, and the remaining participants used the Fitbit Charge 3 model. The data were downloaded from the Fitbit server to the HealthReact study server.^51^ Sleep minutes were excluded, and PA data aggregated to minute-level values were derived from Fitbit (eg, number of steps per minute). Because Fitbit does not provide access to raw accelerometer signals, non-wear time was inferred using summary data. A minute was classified as valid wear time if it included either a non-zero step count or a heart rate reading. This approach aligns with other studies and recommendations in Fitbit-based activity research.^49^^,^^52^^,^^53^ Subsequently, the number of valid minutes (hours, respectively) per day was calculated. The measure of PA was calculated as the average number of steps per valid hour of weartime (ie, total number of daily wake steps divided by hours of weartime on the given day).

*Exercise identity.* Exercise identity was assessed as part of a PA questionnaire that participants completed on the first day of the baseline testing in a laboratory. The 9-item Exercise Identity Scale^54^ was used to measure exercise identity. The internal consistency of the scale (Cronbach's alpha) for the study sample was .94.

*Seasonality (month of the year).* Seasonality was captured using the exact month during which EMA data were collected, spanning from April 2019 to August 2022. Values of LS and PA for January to March were based on data from 3 years (2020, 2021, and 2022); values for months April to August were based on 4-year assessments (years 2019, 2020, 2021, and 2022), and values for September to December on 3 assessments (years 2019, 2020, and 2021).

*Weekly periodicity (days of the week).* Weekly periodicity was assessed using EMA data collected continuously over 1231 days between April 19, 2019, and August 31, 2022, across all participants. This period includes approximately 176 observations for each day of the week (Monday through Sunday), allowing for the examination of weekly patterns in LS and PA (estimated as completely free hours × weekday mean patterns whose submodel is saturated).

### Statistical analyses

Our analyses are based on a dynamic Bayesian model for LS (obtained from daily administered evening survey questions) and PA (obtained as daily per-hour average step counts) with individual-specific random effects. Both LS (also noted as *L* for simplicity to denote the transformed LS variable in model description below) and PA (also noted as *P* for simplicity to denote the transformed PA variable below) are non-normally distributed (departure from normality is induced by the presence of both lower and upper limits for LS and substantial right skew for PA), leading to unstable and statistically improper modeling if taken directly. Instead, they are modeled in a Gaussian model after transformation. All transformations were applied during preprocessing. Specifically, for PA, which was strictly non-negative and highly skewed, we added 1 to each value (to avoid taking the logarithm of zero) and then applied a log transformation. For LS, which was measured on a 0-100 scale, we shifted the data by adding 1 and dividing by 102 to ensure the values fell strictly within the (0,1) interval. We then applied a logit transformation to map the values to the real line. These transformed variables, denoted as *P* (log-transformed PA) and *L* (logit-transformed LS), were used as the dependent variables in our multivariate Gaussian model to obtain components of our bivariate response $(L_{it},P_{it})´$ for *t*th day of observation schedule of *i*th individual. The model is derived from the first principles to respectfully represent the multi-burst structure and possibly bidirectional relationships between LS and PA. Dynamical structure is based on an extension of the VAR model,^55^ allowing for the accommodation of the 4-burst design, separation of between- and within-individual covariate effects, as well as the exercise identity (EXID) covariate effect upon contemporaneous correlation (ie upon correlation of the random disturbances in VAR), and is specified as follows:


$$\left(\begin{array}{c} L_{it}\\ P_{it} \end{array})=(\begin{array}{c} \mu_{Q,it}\\ \mu_{P,it} \end{array})+(\begin{array}{c} m_{L,i}\\ m_{P,i} \end{array})+(\begin{array}{cc} \beta_{LL} & \beta_{LP}\\ \beta_{PL} & \beta_{PP} \end{array}).(\left(\begin{array}{c} L_{i,t-1}\\ P_{i,t-1} \end{array})-(\begin{array}{c} \mu_{L,it}\\ \mu_{P,it} \end{array}\right))+(\begin{array}{c} \varepsilon_{L,it}\\ \varepsilon_{P,it} \end{array}\right)$$

where

-
$\left(\begin{array}{c} \varepsilon_{L,it}\\ \varepsilon_{P,it} \end{array})∼N(\left(\begin{array}{c} 0\\ 0 \end{array}),(\begin{array}{cc} \sigma_{L}^{2} & \sigma_{L}.\sigma_{P}.\rho ({\alpha ;\mathrm{EXID}}_{i})\\ \sigma_{L}.\sigma_{P}.\rho ({\alpha ;\mathrm{EXID}}_{i}) & \sigma_{P}^{2} \end{array}\right)\right)$ are random disturbances allowing for contemporaneous correlation (ρ).
- The contemporaneous correlation is allowed to depend on EXID to explore the extent to which EXID moderates the relationship between *L* and *P*. Obviously, the EXID influence has to be (strictly monotone) link-transformed in order to stay in the legal correlation range. In particular, we use the Fisher *z* transformation^56^ as the link and hence have: $\rho ({\alpha ;\mathrm{EXID}}_{i})=\frac{\;\text{exp}\;(2\times (\alpha_{0}+\alpha_{1}.{\mathrm{EXID}}_{i}))-1}{ex(2\times (\alpha_{0}+\alpha_{1}.{\mathrm{EXID}}_{i}))+1}$ with $\alpha_{0},\;\alpha_{1}$ parameters to be estimated.
-
m terms are individual-specific uncorrelated random effects $m_{L,i}∼N(0,\tau_{L}^{2})$, $m_{P,i}∼N(0,\tau_{P}^{2})$. These are important in allowing for correlation among one person’s outcomes among different bursts. Also, their exchangeable structure allows for the desirable shrinkage of Stein type,^57^ usually encountered in hierarchical modeling.^58^
- Covariate effects influencing the means of *L* and *P* are incorporated into μ terms, namely $\mu_{L,it}=\delta_{L0}+\sum_{k=1}^{K}\delta_{Lk}.X_{ik}$, $\mu_{P,it}=\delta_{P0}+\sum_{k=1}^{K}\delta_{Pk}.X_{ik}$.
- Choice of both continuous and categorical *X* covariates gives a general linear predictor structure analogous to analysis of covariance structure. In particular, we use categorical variables gender, location (MSR vs SBR), education (university vs lower than university), SES (3 levels giving 2 indicator variables with effects parametrized as differences from SES “below average”), month (11 indicator variables with effects parametrized as differences from January), weekday (6 indicator effects with effects parametrized as differences from Monday) plus 2 continuous variables: day in the study and age. These covariates were modeled as predictors of the mean levels of LS and PA, thereby accounting for systematic between-person and temporal variation. However, they were not specified as moderators of the within-person contemporaneous association. The only variable formally modeled as a moderator of this association was exercise identity, which was incorporated into the covariance structure of the VAR disturbances.
- It is vital that our model allows for mean periodicities at 2 levels (weekly and annual) that should not be confused with individual dynamics.
-
$\left(\begin{array}{cc} \beta_{LL} & \beta_{LP}\\ \beta_{PL} & \beta_{PP} \end{array}\right)$ is the Markovian transition matrix (containing first-order autoregressive and cross-regressive coefficients), allowing for lagged effects both within the same variable as well as between *L* and *P*.

The model parameters are: regression coefficients (δs), dynamic parameters (ie, VAR submodel parameters) or autoregressive coefficients (βs), contemporary correlation parameters ($\alpha_{0},\;\alpha_{1}$), random VAR disturbance standard deviations (σs), random effects’ standard deviations (τs), and random effects themselves (ms). The model is fitted within Bayesian framework, using rather flat priors independent among the parameters. In particular, we used uniform priors for the dynamic (VAR) parameters within the constraints dictated by stationarity, ie, they are restricted to the (−1,1) interval, uniform priors for the regression coefficients on covariates (with no a priori range restriction), and the half-normal priors for the standard deviations.^59^ Concurrent correlations were restricted to the (−1,1) interval due to the Fisher *z*-transform parametrization mentioned above (and no restrictions on coefficients $\alpha_{0},\;\alpha_{1}$). We checked the sensitivity to priors by manipulating the prior standard deviations from half to double of the original and found very good agreement with the results reported in Tables 2-4 (correlation coefficients 0.9999 and higher). The occasional missing data (and first observations in a burst) are treated as MAR (missing at random),^60^ and hence the analysis is conducted on observed data only, as we have no reliable information about the mechanisms of missingness. We use Hamiltonian Monte Carlo simulations in Stan^61^ for computations. Obvious and practically important implication of the Bayesian, fully probabilistic formulation of the model is that all model parameters are estimated simultaneously in 1 step without any ad hoc fixes and that the estimates fully acknowledge uncertainties propagating through the relationship among parameters. To improve convergence, we standardize the covariates and rescale the resulting coefficients (reporting them on original, not standardized scale). Non-convergence was not an issue in this case, with no divergent transitions after warmup, Rhats essentially 1 (the largest Rhat for parameters is 1.0098).

**Table 2.** Bayesian VAR, unified model with moderation of the concurrent relationship—predicting transformed life satisfaction (regression coefficients expressed without standardization).

			95% Credible interval		
Variable	Posterior *M*	Posterior SD	Lower limit	Upper limit	ESS
**Intercept**	−0.4327	0.3109	−1.0533	0.1690	813
**Cumulative days in study**	0.0017	0.0007	0.0002	0.0031	7824
**Gender (male)**	0.0910	0.0794	−0.0673	0.2502	584
**Age**	0.0151	0.0031	0.0089	0.0213	675
**Location (Moravian-Silesian Region)**	−0.0917	0.0835	−0.2610	0.0722	753
**Weekday 2 (Tuesday)**	0.2556	0.0414	0.1758	0.3378	4023
**Weekday 3 (Wednesday)**	0.2017	0.0423	0.1206	0.2855	3372
**Weekday 4 (Thursday)**	0.2202	0.0417	0.1387	0.3006	3735
**Weekday 5 (Friday)**	0.3623	0.0418	0.2789	0.4424	3666
**Weekday 6 (Saturday)**	0.7257	0.0536	0.6216	0.8292	4322
**Weekday 7 (Sunday)**	0.4406	0.0414	0.3587	0.5221	3803
**Month 2 (February)**	−0.0556	0.0676	−0.1852	0.0789	1827
**Month 3 (March)**	−0.2274	0.0713	−0.3625	−0.0878	1882
**Month 4 (April)**	−0.0112	0.0706	−0.1532	0.1250	1712
**Month 5 (May)**	−0.0034	0.0610	−0.1187	0.1158	2286
**Month 6 (June)**	0.0825	0.0640	−0.0447	0.2106	1654
**Month 7 (July)**	0.2484	0.0694	0.1127	0.3861	1391
**Month 8 (August)**	0.1270	0.0689	−0.0091	0.2637	1496
**Month 9 (September)**	0.0225	0.0614	−0.0983	0.1431	2255
**Month 10 (October)**	−0.0270	0.0637	−0.1483	0.1023	1949
**Month 11 (November)**	−0.0903	0.0715	−0.2287	0.0571	1280
**Month 12 (December)**	0.0422	0.0688	−0.0892	0.1739	1533
**Education (university education)**	−0.0273	0.0824	−0.1877	0.1329	675
**SES (average)**	0.4257	0.2643	−0.0805	0.9497	938
**SES (above average)**	0.5129	0.2766	−0.0197	1.0550	954

Abbreviations: SES, socioeconomic status; *M*, mean; SD, standard deviation; ESS, effective sample size.

**Table 3.** Bayesian VAR, unified model with moderation of the concurrent relationship—predicting transformed physical activity (regression coefficients expressed without standardization).

			95% Credible interval		
Variable	Posterior *M*	Posterior SD	Lower limit	Upper limit	ESS
**Intercept**	6.1004	0.1039	5.8999	6.2984	1561
**Cumulative days in study**	−0.0019	0.0003	−0.0025	−0.0013	7801
**Gender (male)**	0.0840	0.0269	0.0305	0.1358	993
**Age**	0.0069	0.0011	0.0048	0.0090	1122
**Location (Moravian-Silesian Region)**	−0.0183	0.0274	−0.0707	0.0358	1269
**Weekday 2 (Tuesday)**	0.0914	0.0175	0.0569	0.1245	4510
**Weekday 3 (Wednesday)**	0.0490	0.0175	0.0146	0.0829	4193
**Weekday 4 (Thursday)**	0.0713	0.0176	0.0365	0.1060	4699
**Weekday 5 (Friday)**	0.0752	0.0180	0.0395	0.1106	4458
**Weekday 6 (Saturday)**	0.1676	0.0220	0.1239	0.2094	5004
**Weekday 7 (Sunday)**	0.0388	0.0175	0.0051	0.0723	4375
**Month 2 (February)**	0.0474	0.0282	−0.0077	0.1016	2066
**Month 3 (March)**	0.1074	0.0298	0.0506	0.1663	2224
**Month 4 (April)**	0.1086	0.0292	0.0521	0.1672	2035
**Month 5 (May)**	0.1346	0.0260	0.0840	0.1856	2650
**Month 6 (June)**	0.2298	0.0270	0.1777	0.2836	2156
**Month 7 (July)**	0.2086	0.0281	0.1537	0.2631	1885
**Month 8 (August)**	0.1952	0.0281	0.1387	0.2507	1920
**Month 9 (September)**	0.1985	0.0254	0.1486	0.2497	2356
**Month 10 (October)**	0.0846	0.0272	0.0323	0.1380	2159
**Month 11 (November)**	0.0797	0.0297	0.0211	0.1367	1936
**Month 12 (December)**	0.0520	0.0287	−0.0047	0.1083	2079
**Education (university education)**	0.0051	0.0279	−0.0477	0.0605	1184
**SES (average)**	−0.0091	0.0885	−0.1823	0.1669	1498
**SES (above average)**	0.0155	0.0937	−0.1678	0.2013	1402

Abbreviations: VAR, vector autoregressive; SES, socioeconomic status; *M*, mean; SD, standard deviation; ESS, effective sample size.

**Table 4.** Bayesian VAR, unified model variance/covariance parameters, and dynamic parameters.

Variance and covariance parameters					
			95% Credible interval		
Parameter	Posterior *M*	Posterior SD	Lower limit	Upper limit	ESS
**sigma1** ${(\delta }_{L0}$)	1.2342	0.0052	1.2245	1.2442	6432
**sigma2** $\left(\delta_{P0}\right)$	0.5855	0.0024	0.5808	0.5902	8039
**tau1** ($\tau_{L}^{2})$	1.1847	0.0294	1.1292	1.2428	6163
**tau2** ($\tau_{P}^{2}$)	0.3870	0.0106	0.3668	0.4081	5197
**alpha0** ($\alpha_{0})$	0.0872	0.0042	0.0791	0.0951	7216
**alpha1** ($\alpha_{1})$	0.0105	0.0025	0.0056	0.0156	4720

Dynamic parameters						
				95% Credible interval		
	Parameter	Posterior *M*	Posterior SD	Lower limit	Upper limit	ESS
**VAR effect of previous day’s LS on next day’s LS**	$\beta_{LL}$	0.3941	0.0055	0.3831	0.4051	5973
**VAR effect of previous day’s LS on next day’s PA**	$\beta_{LP}$	−0.0013	0.0117	−0.0245	0.0215	6634
**VAR effect of previous day’s PA on next day’s LS**	$\beta_{PL}$	0.0011	0.0026	−0.0041	0.0061	6413
**VAR effect of previous day’s PA on next day’s PA**	$\beta_{PP}$	0.3158	0.0059	0.3040	0.3271	6611

Abbreviations: VAR, vector autoregressive; LS, life satisfaction; PA, physical activity; *M*, mean; SD, standard deviation; ESS, effective sample size.

## Results

The analysis included data from adults (*N* = 1314) with a mean age of 38.09 (SD = 12.55). Women made up 46.3% of the sample, and 56.8% of the sample self-identified as active runners. The description of the study sample can be found in Table 1. The Bayesian VAR model^55^ with moderation of the concurrent within-person relationship (by exercise identity) provided several insights into the relationship between daily LS and PA.

### Individual effects on transformed life satisfaction

**Figure 1.**
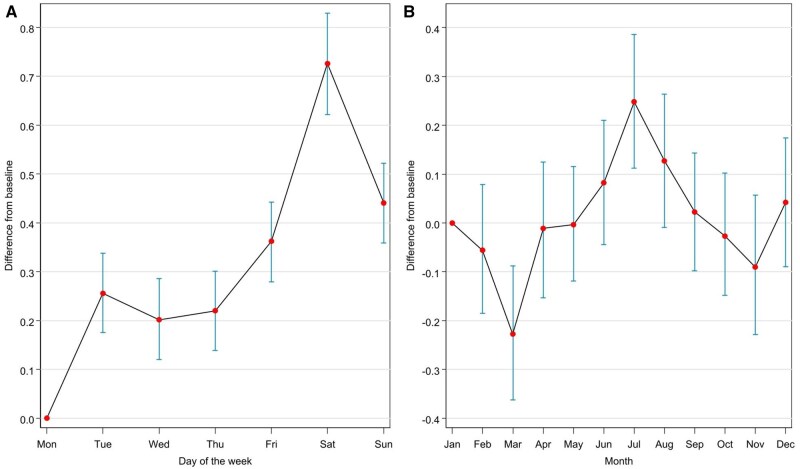
Graphic representation of the regression coefficients representing temporal trends in LS (A: weekly periodicity; B: seasonality). LS, life satisfaction.

The results of the individual effects on transformed LS are presented in Table 2. As can be seen, the intercept had a posterior mean of −0.4327, with a 95% credible interval ranging from −1.0533 to 0.1690, indicating no credible baseline effect on LS. The effect for cumulative days in the study, however, showed a small but important positive effect (posterior mean = 0.0017, 95% CI [0.0002, 0.0031]), suggesting that LS slightly increased over the course of the 12-month study. March showed a credible negative effect on LS (posterior mean = −0.2274, 95% CI [−0.3625, −0.0878]), while July had a credible positive effect (posterior mean = 0.2484, 95% CI [0.1127, 0.3861]). Other months did not show important effects. At the same time, credible positive effects were observed for all weekdays compared to Monday, with the strongest positive effects on Saturday (posterior mean = 0.7257, 95% CI [0.6216, 0.8292]) and Sunday (posterior mean = 0.4406, 95% CI [0.3587, 0.5221]). The weekly periodic (days of the week) and seasonal (month of the year) components for LS are depicted in Figure 1 in graphs A and B, respectively.

In terms of sociodemographic variables, the effects for gender (posterior mean = 0.0910, 95% CI [−0.0673, 0.2502]), location (posterior mean = −0.0917, 95% CI [−0.2610, 0.0722]), and education (posterior mean = −0.0273, 95% CI [−0.1877, 0.1329]) were not meaningful. However, age had a credible positive effect on LS (posterior mean = 0.0151, 95% CI [0.0089, 0.0213]), indicating that older participants reported higher LS. Higher SES levels showed positive effects on LS; however, these effects were small.

### Individual effects on transformed physical activity

**Figure 2.**
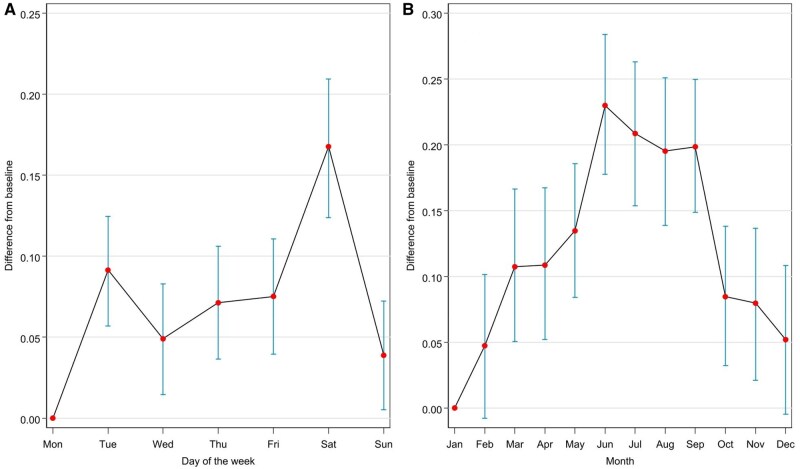
Graphic representation of the regression coefficients representing temporal trends in PA (A: weekly periodicity; B: seasonality). PA, physical activity.

The results of the individual effects on transformed PA are presented in Table 3. As can be seen, the intercept had a posterior mean of 6.1004, with a 95% credible interval ranging from 5.8999 to 6.2984, indicating a credible effect of baseline level of PA. The effect for cumulative days in the study showed a small but important negative effect (posterior mean = −0.0019, 95% CI [−0.0025, −0.0013]), suggesting a slight decrease in PA over the course of the study. The regression coefficients for the weekly periodicity (days of the week, graph A) and seasonality (month of the year, graph B) effects for PA are depicted in Figure 2, indicating important positive effects for all weekdays compared to Monday, with the strongest positive effects on Saturday (posterior mean = 0.1676, 95% CI [0.1239, 0.2094]) and Tuesday (posterior mean = 0.0914, 95% CI [0.0569, 0.1245]). In terms of seasonality effects, several months showed credible positive effects on PA, including March (posterior mean = 0.1074, 95% CI [0.0506, 0.1663]), April (posterior mean = 0.1086, 95% CI [0.0521, 0.1672]), May (posterior mean = 0.1346, 95% CI [0.0840, 0.1856]), June (posterior mean = 0.2298, 95% CI [0.1777, 0.2836]), July (posterior mean = 0.2086, 95% CI [0.1537, 0.2631]), August (posterior mean = 0.1952, 95% CI [0.1387, 0.2507]), and September (posterior mean = 0.1985, 95% CI [0.1486, 0.2497]). Other months did not show important effects. In terms of sociodemographic variables, males (posterior mean = 0.0840, 95% CI [0.0305, 0.1358]) and older participants (posterior mean = 0.0069, 95% CI [0.0048, 0.0090]) had higher levels of PA, whereas no meaningful effects emerged for location (residency in region with high vs low air pollution), education, or SES.

### The dynamics of the life satisfaction and physical activity relationship

The variance and covariance parameters and dynamic model parameters are presented in Table 4. The analysis revealed important positive VAR effects for both LS ($\beta_{LL}$ posterior mean of 0.3941, with a 95% credible interval ranging from 0.3831 to 0.4051) and PA ($\beta_{PP}\;$posterior mean of 0.3158, with a 95% credible interval ranging from 0.3040 to 0.3271), indicating that the previous day’s levels of LS and PA meaningfully predicted their respective next day’s future levels. The cross-lagged effect from the previous day’s LS on the next day’s PA was not important ($\beta_{LP}\;$posterior mean = −0.0013, 95% CI [−0.0245, 0.0215]), neither was the cross-lagged effect from the previous day’s PA on the next day’s LS ($\beta_{PL}\;$posterior mean = 0.0011, 95% CI [−0.0041, 0.0061]).

The significant covariance between the disturbance terms indicated a meaningful within-person contemporaneous association between LS and PA ($\alpha_{0}\;$posterior mean of 0.0872, with a 95% credible interval ranging from 0.0791 to 0.095), suggesting that on days when individuals engaged in higher levels of PA throughout the day, they also reported higher levels of LS in the evening questionnaire. This relationship was moderated by exercise identity ($\alpha_{1}\;$posterior mean of 0.0105, with a 95% credible interval ranging from 0.0056 to 0.0156), indicating that individuals with a stronger exercise identity experienced a more pronounced positive association between PA and LS.

## Discussion

The present study utilized a Bayesian VAR model to explore the dynamic interplay between LS and daily PA. Previous literature has suggested a positive association between LS and PA; however, few studies have explored the temporal ordering of this association and the links to other internal and external contextual variables that could impact this relationship. The design of the present study afforded probing of the reciprocal association between LS and PA in the context of temporal factors and long-term trends. The findings highlight the importance of temporal factors, such as the day of the week and specific months within a year, in influencing PA and LS levels, while the dynamic parameters indicated substantial autoregressive effects for both LS and PA. Additionally, at the within-person level, more PA on a given day was associated with higher LS reported in the evening questionnaire on the same day, but more so for individuals with a stronger sense of exercise identity. Given the study design (PA assessed throughout the day and LS at the end of the day between 20:00 and 22:00), the modeled relationship provides a temporal order for the association within a day.

### Temporal factors and longitudinal trends

This is the first study to demonstrate in 1 analysis that both the day of the week and specific month of the year are significantly associated with PA and LS. PA levels were notably higher on weekends, particularly on Saturdays, and LS was also higher on weekends, with the strongest effect observed on Saturdays. This is in line with other studies and suggests that weekends provide more opportunities for engaging in PAs^62–64^ and experiencing higher LS,^65–68^ likely due to increased leisure time^69^ and reduced work-related stress.^70^

Also, PA was higher in June and July, indicating seasonal variation with warmer months encouraging more PA (likely due to better weather conditions and longer daylight hours). Conversely, March had a significant negative effect on LS, possibly due to lingering winter conditions and less favorable weather.^71^^,^^72^ While no significant negative effects were observed for January or February, the March decline in LS may reflect a mismatch between psychological expectations of seasonal improvement and continued unfavorable weather conditions. Prior research has shown that springtime, particularly March and April, is paradoxically associated with increased depressive symptoms and suicide risk,^73^ likely due to rapid changes in photoperiod, behavioral expectations, and unfulfilled anticipation of improved conditions.^74^^,^^75^ These transitional stressors may make March a uniquely vulnerable period for subjective well-being. Given that EMA data collection occurred between April 2019 and August 2022, meaning that seasonal and monthly effects are based on observations from 2 to 3 years, another possible reason for lower LS in March is the first COVID-19 lockdown, a period widely recognized for its negative impact on LS and mental health (eg,^76^) The first lockdown was implemented in the Czech Republic in March 2020 and may have contributed to the overall lower LS in March (mean of LS in March 2020, 2021, and 2022). Across the 12-month study, PA slightly decreased and LS slightly increased (see the cumulative days in Tables 2 and 3). These trends could reflect adaptation or changes in participants’ routines and perceptions over the study period or reflect decreasing level of motivation to sustain PA over time.^28^^,^^29^

### Dynamic relationships

The significant autoregressive effects for both LS and PA suggest that individuals’ previous levels of LS and PA are strong predictors of their future levels. This aligns with existing literature indicating that, in the absence of intervening factors, both LS and PA are driven by past behavior; however, this study also highlights how weekly periodicity and seasonality are likely to impact changes in PA and LS over time. We failed to find support for lagged associations between LS and PA. This is contrary to research by van Woudenberg et al^25^ who demonstrated reciprocal effects of PA and happiness in adolescents, showing that PA levels can predict future happiness and vice versa. Nonetheless, our research supported a significant within-person contemporaneous association between LS and PA, highlighting the immediate positive impact of PA on LS. This finding is consistent with previous research demonstrating that engaging in more PA on a given day can boost mood and overall LS (eg,^19^^,^^21^^,^^22^^,^^77^)

Our finding of a robust contemporaneous within-person association between daily PA and LS, but no significant cross-lagged effects, suggests that the impact of PA on LS may be short-lived and time-sensitive, consistent with affective regulation models.^78^ That is, individuals may experience boosts in LS on days when they are more physically active, but these effects may not persist or accumulate across days in the absence of repeated engagement in PA. This pattern has practical implications for interventions aiming to improve LS through PA that may need to focus on supporting frequent and consistent activity, rather than relying on delayed or cumulative psychological benefits.

Furthermore, the moderation of this association by exercise identity underscores the psychological context in which PA occurs. The stronger the personal alignment with PA (ie, “seeing oneself as an exerciser”), the more likely that same-day activity contributes meaningfully to LS. This reflects a self-congruence mechanism, whereby behaviors that reinforce one’s identity are experienced as more satisfying. These dynamics point toward the potential utility of identity-informed behavioral interventions, not just promoting PA behaviorally, but cultivating a self-concept in which PA is meaningful and rewarding. Future research should examine whether incorporating strategies supporting reflective processes (eg, enhancing self-efficacy or self-regulation skills) and reflexive processes (eg, strengthening habit) could serve to enhance exercise identity, thus boosting the effects of PA on LS. It should be noted that other demographic and contextual variables (eg, age, gender, SES, education, seasonality, and residency status) were modeled as covariates influencing the mean levels of LS and PA, rather than as moderators of the within-person contemporaneous PA-LS association. This distinction highlights exercise identity as the unique variable tested for moderation in this study, while pointing to opportunities for future work to extend moderation analyses to other individual and contextual factors. Additionally, although baseline activity status (runner vs. inactive control) could be viewed as a potential moderator, we did not include it in the moderation analysis because it is related to both exercise identity and objectively measured PA. Exercise identity was prioritized as it aligns more closely with the theoretical causal mechanism of the PA-LS link, that is, the idea that PA exerts stronger effects on LS when it becomes part of one’s self-concept. Future research could test activity status directly to further disentangle its unique role relative to exercise identity and PA (ideally employing a specialized statistical design).

Finally, the co-patterning of PA and LS with temporal cycles (weekdays, seasons) further suggests that the LS-PA relationship is not fixed but context-dependent and dynamic, shaped by external cues and routines. This highlights the value of longitudinal and ecologically grounded study designs like the one used here, which allow for uncovering these time-sensitive interdependencies that may be obscured in cross-sectional or retrospective designs. While this points to the importance of considering contextual and temporal variation, we did not formally test whether the strength of the PA-LS relationship varied across these cycles. Our findings indicate co-patterning with temporal cycles, but not moderation. Future work (based on specific statistical designs) is needed to examine such moderation explicitly.

### Associations with sociodemographic factors

Age was positively related to both PA and LS, suggesting that older individuals tend to engage more in PA and report higher LS, which could be due to more established routines and fewer work- or family-related obligations.^34^ Although epidemiological studies indicate decreases in PA with age,^79^^,^^80^ some longitudinal investigations show that PA does not decline uniformly in all, but that factors such as socioeconomic disadvantage, high BMI, or poor health play a role.^81^ The increasing age-related trend in PA in the current study may thus reflect the unique nature of the sample, comprised of overall healthy adults and active runners (56.8%).

The age-related trend in LS is in line with a recent meta-analytic review of longitudinal studies demonstrating that LS declined during adolescence, increased slightly until the age of 70, and decreased afterwards until the age of 96.^33^ Males had higher PA levels, but gender did not have a significant effect on LS, which is in line with observations from longitudinal studies.^33^ In terms of education and SES, there were no strong effects on either PA or LS, but future studies should probe these associations with more diverse samples. There was also no effect of residency status on either PA or LS, which is in contrast with previous studies that have demonstrated relationship of exposure to air pollutants and indicators of well-being or PA level reflected differences in air pollution status between the Moravia-Silesia and South Bohemia regions. Previous studies have demonstrated links between air pollution and indicators of well-being.^27^^,^^28^^,^^82^ There is also evidence suggesting that persistent air pollution discourages PA, leading to negative consequences for physical and mental health (eg,^83^). One possible explanation of this discrepancy is that residency status may not adequately capture individual-level air pollution exposure. Future studies should incorporate refined measurement of air pollution, including direct measurements of pollutant concentrations or individual exposure, ideally capturing daily variation in air quality alongside time-specific indicators of PA and well-being.

### Study limitations

Despite the valuable insights provided by this study, several limitations should be acknowledged. First, although both runners and non-runners were included in the sample, the overall activity level was rather high. This may limit the generalizability of the findings to less active populations. Future research should aim to include a more diverse range of activity levels to better understand the relationship between LS and PA across different segments of the population.

Second, we assumed that data were MAR, which is a widely accepted approach in the analysis of intensive longitudinal data, particularly in the absence of strong a priori knowledge about the mechanisms underlying missingness.^84^ This assumption was necessitated by the lack of auxiliary variables and the complexity of the study design, which precluded more detailed modeling of missingness processes. In our Bayesian dynamic VAR framework, missing data are accommodated through full-information posterior estimation, which allows for uncertainty due to missingness to be directly incorporated into all parameter estimates. This approach reduces the bias commonly associated with ad hoc imputation methods or empirical modeling that lacks theoretical justification. While we recognize that the MAR assumption may not fully hold, we consider this modeling strategy a conservative and pragmatic choice given the current data structure. Future research could benefit from more formal modeling of missingness mechanisms, such as shared parameter models or pattern-mixture models,^85^ particularly if guided by theoretical or expert-informed assumptions about why data are missing. Nonetheless, sensitivity analyses (available upon request) indicated that our main conclusions were robust under different missing data assumptions.

Third, we did not account for differences in other variables known to differentially impact LS, such as BMI.^86^ Although there were few overweight and obese individuals in the sample, BMI can influence both PA and LS.^81^ Future research should control for BMI and other relevant variables to provide a more comprehensive understanding of the factors influencing LS. Fourth, residency status (region of high or low air pollution) was used as a proxy for air pollution exposure, without including daily air pollution measures or individual-level exposure to specific pollutants such as PM_2.5_, PM_10_, or NO_2_. This approach may not accurately capture the day-to-day variations in air pollution exposure that could affect the relationship between PA and LS, or it may overlook more precise estimates of long-term exposure to air pollutants, as both regions are relatively large and likely to contain substantial within-region variability in air pollution levels. Future studies should incorporate more precise measures of air pollution, such as air quality indices from wearable mobile sensors. Additionally, longitudinal studies with more diverse samples are needed to confirm these findings and extend them to different populations. Understanding the long-term effects of PA on LS and identifying factors that can enhance this relationship will be crucial for developing comprehensive well-being interventions.

### Implications for interventions

In spite of these limitations, these findings have important implications for interventions aimed at enhancing LS through PA. The significant influence of weekdays and months on PA and LS suggests that interventions should be tailored to these temporal patterns. For example, promoting PA on weekends, when people are more likely to engage in such activities, could be particularly effective, or supporting them in breaking up sedentary periods on workdays. Seasonal variations also indicate that interventions might need to be adjusted throughout the year, with more emphasis on encouraging outdoor activities during warmer months and providing additional support for fitting in doable activities throughout the day during winter months.^87^^,^^88^ For example, just-in-time adaptive interventions (or JITAIs; eg,^89^^,^^90^) can leverage real-time data to adapt to an individual’s current context such that, if a person is less active on weekdays, the intervention could provide additional encouragement or suggest shorter, more manageable activities during these times. Also, given the non-significant cross-lagged effects, it may be more appropriate to focus on providing continuous support rather than expecting immediate shifts in well-being. This could involve regular check-ins and adjustments based on the individual’s ongoing activity levels and feedback.

To counteract the slight decrease in PA over time observed in our study, JITAIs could incorporate features that help sustain motivation, such as gamification elements, social support networks, and personalized challenges that evolve based on the user’s progress and preferences, as well as promote a strong exercise identity. The moderation effect of exercise identity on the PA-LS association suggests a critical pathway for tailoring interventions. For individuals with a strong exercise identity, the psychological benefits of PA may be more immediate and reinforcing. For these individuals, interventions might focus on maintaining motivation, preventing burnout, or supporting consistency during times when external constraints limit activity (eg, injury, work, seasonal changes). However, fostering exercise identity among individuals with initially low identification with PA presents a challenge. Identity-related constructs tend to be stable and deeply rooted in self-concept.^91^ Changing one’s identity is not straightforward and typically requires sustained behavior change, social reinforcement, and alignment with personal values.^92^^,^^93^

Therefore, interventions might target identity development through diverse mechanisms spanning both reflective processes (self-monitoring, goal setting, and values clarification to help individuals recognize the personal significance of PA) as well as reflexive processes (habit formation through regular activity cues and rewarding experiences, building toward automaticity). Social identity mechanisms for building exercise identity may include the promotion of group-based activities or encouraging exercise in contexts where PA is normative and valued. Finally, narrative-based approaches, where participants reflect on and reframe their life stories to incorporate PA as part of who they are becoming, could be utilized.^94^ The utilization of these strategies in the context of JITAIs, which dynamically respond to individual behavior and context, may mean, for instance, providing tailored feedback or motivational prompts when users show signs of disengagement or when opportunities for identity-aligned activity (eg, community events, preferred routines) emerge. Over time, reinforcing small identity-consistent actions can lead to a more internalized sense of being “an active person,” enhancing both adherence and well-being.^40^^,^^95^^,^^96^

In sum, interventions that are temporally aware, context-sensitive, and identity-informed may be especially effective in promoting sustained PA and its downstream benefits on LS. Future work should explore the feasibility and effectiveness of these strategies in real-world settings and across diverse populations.

## Conclusion

The results highlight the significant influence of temporal factors, such as weekdays and months, on PA and LS. Unlike residency (living in a region of high or low air pollution), factors including age, gender, and exercise identity also play important roles. The dynamic parameters confirm the strong autoregressive effects of LS and PA, as well as a robust within-person contemporaneous association between these variables, indicating that PA on a given day was positively associated with LS reported in the evening on the same day.

## Data Availability

The data used in this study are available upon request as per the data sharing policy of the 4HAIE project at https://haie-lerco.cz/en/data-en/.

## Open science transparency statements

Study registration: This study was not registered. Analytic plan registration: The analytic plan was not pre-registered. Availability of data: The data used in this study are available upon request as per the data sharing policy of the 4HAIE project at https://haie-lerco.cz/en/data-en/. Availability of analytic code: The analytic code used in this study is available upon request from mbrabec@cs.cas.cz. Availability of materials: The study protocol papers and data codebooks are available at https://haie-lerco.cz/en/data-en/. Other materials used in this study are not publicly available.
